# Battling the COVID-19 infodemic in an Irish context: the role of iHealthFacts

**DOI:** 10.12688/hrbopenres.13174.1

**Published:** 2020-11-09

**Authors:** Marina Zaki, Declan Devane, Thomas Conway, Sandra Galvin, Nikita Burke, Elaine Finucane

**Affiliations:** 1 School of Nursing and Midwifery, National University of Ireland Galway, Galway, Ireland; 2 Evidence Synthesis Ireland, National University of Ireland Galway, Galway, Ireland; 3 Health Research Board – Clinical Research Facility Galway, National University of Ireland, Galway, Galway, Ireland

**Keywords:** COVID-19, SARS-CoV-2, Infodemic, Fake News, Health Claims, Health Facts, Social Media, Fact Check

## Abstract

On the 11 ^th^ of March 2020, the World Health Organisation (WHO) declared a global pandemic due to the SARS-CoV-2 virus, which causes coronavirus disease 2019 (COVID-19). This was one month after Dr. Tedros Adhanom Ghebreyesus, Director-General of the WHO declared that we are also fighting an ‘infodemic’. The WHO has described an infodemic as an *“over-abundance of information – some accurate and some not – that makes it hard for people to find trustworthy sources and reliable guidance when they need it”*. iHealthFacts.ie is an Irish resource where the public can quickly and easily check the credibility and reliability of health claims circulating on social media. Unreliable claims can lead to poorly informed health choices. iHealthFacts is an initiative that supports the public to think critically about health claims and make well-informed choices. Here, we describe the role iHealthFacts plays in providing reliable information to the public and offer reflections from those involved in launching this initiative during a pandemic.

## Disclaimer

The views expressed in this article are those of the authors. Publication in HRB Open Research does not imply endorsement by the Health Research Board of Ireland.

Unreliable claims can lead to poorly informed choices, under- or over-use of treatments, unnecessary waste, and human suffering ( Chalmers *et al.*, 2018; Gottlieb, 2000). This poses a significant risk to health and healthcare. This risk to public health is illustrated starkly by, but not limited to, the coronavirus disease 2019 (COVID-19) pandemic, which has been accompanied by what the World Health Organization (WHO) has called an ‘infodemic’ - an over-abundance of information – some accurate and some not – that makes it hard for people to find trustworthy scientific information when they need it.

Although the COVID-19 pandemic highlighted the infodemic, the outbreak of misinformation (and disinformation) is commonplace in today’s communication society. Every day health claims are made about the safety and efficacy of vaccinations, nutrition and the effects of specific treatments ( Chang, 2018; Verhagen *et al.*, 2010). The spread of misinformation is not only potentially harmful to people’s physical health but also their mental health. Many of the claims circulating on social media foster fearmongering, and induce stress and anxiety amongst the public, leading to significant human suffering and may even result in loss of life ( Gao *et al.*, 2020; Rathore & Farooq, 2020).

Efforts to assess the reliability of health claims have come from several sources: including the media (e.g., thejournal.ie), individual science communicators and health care organisations (the WHO and the Centres for Disease Control and Prevention). Tackling fake news and preventing the spread of unsubstantiated health claims, especially during a public health crisis, requires healthcare professionals, researchers, scientists, policymakers, and members of the public to work together to support people to think critically about health claims and make well-informed choices.

## The role of social media

Social media plays a critical role in disseminating health information to the public ( Ahmad & Murad, 2020). During the initial phases of the novel coronavirus epidemic (before the announcement of the COVID-19 pandemic), social media users noticed an influx of health claims. Social media platforms were bombarded with new claims connected to the detection, treatment or prevention of COVID-19. These were often in the form of ‘remedies’ or techniques that could apparently detect, prevent or cure COVID-19. Examples included: drinking alcohol to prevent COVID-19, detecting the virus by thermal scanners, ingesting bleach to kill the virus and the ability to test for the presence of COVID-19 by holding one’s breath for more than ten seconds ( Freckelton, 2020).

Timely identification of potentially harmful health claims is important. As noted by Dr. Tedros of the WHO, health claims about COVID-19 often appeared to spread faster than the virus itself, posing a significant challenge. The onus is not only on researchers, scientists and clinicians to try to combat these claims, but on all social media users to begin to question the reliability of health claims. The message for social media users is simple: when confronted with a health claim, do not share further until your decision to share is informed by the source of the claim, the reason is it being sent, how the treatment was assessed and if the claim is supported by reliable evidence.

The WHO have emphasised the importance of technical risk communication and social media teams in responding to the sudden surge of claims and rumours about SARS-CoV-2 ( WHO Novel Coronavirus Situation Report – 13). The WHO and its partners worked to identify and address common claims that may have impacted public health negatively. Social media channels including Weibo, Twitter, Facebook, Instagram, LinkedIn, Pinterest, and TikTok were used by the WHO to promote their message of reducing the harm from misinformation. While global efforts to tackle fake news were evident, a focus on answering claims relating directly to the Irish public was needed.

## iHealthFacts team and process

iHealthFacts.ie is an Irish initiative based at the National University of Ireland Galway, whose aim is to provide clear and reliable information to the public. Our goal is to help members of the public to think critically about health claims and to make well-informed choices. iHealthFacts, funded by the Health Research Board (Ireland), is a collaboration between Evidence Synthesis Ireland, Cochrane Ireland, the Health Research Board – Trials Methodology Research Network (HRB-TMRN) and NUI Galway.

The multi-disciplinary iHealthFacts team comprises researchers, clinicians, scientists, bacteriologists, General Practitioners, pharmacists, epidemiologists, biologists, endocrinologists, obstetricians, statisticians, public and patient representatives, a health journalist, and a social media coordinator – spanning several universities across the island of Ireland. Each bring their own unique set of skills and expertise to the processes underpinning iHealthFacts.

iHealthFacts answers health claim questions submitted by the public using robust processes. We prioritise submitted health claim questions based on: 1) their importance to the public, achieved by ranking the submitted health claims in order of popularity; 2) a health claims’ potential for adverse harm to the public.

Each claim is researched systematically using relevant experts and a response written with a focus on making the information understandable and accessible. The process begins by assigning a prioritised claim to a named member of our research team who undertakes the preliminary research and writes the first draft of the health claim check.

We try to locate the source material of the claim for accuracy and elaboration (e.g. social media posts). We then search for evidence to support or refute the claim. We search for systematic reviews of studies to help us inform decisions. If these are not available, we search for individual studies. Where appropriate, we also search for statements from reputable health agencies. For complex claims, other members of the research team may provide additional research support.

Once a health claim has been researched and an ‘answer’ drafted, a second named researcher reviews it independently. The answer is then reviewed by at least one healthcare specialist and public representative from of our team of Evidence Advisors before final review by our Health Journalist.

We recognise that the evidence basis evolves over time and appreciate the need for, and value of, post-publication clarifications by highlighting when new evidence is found, the claim’s ‘answer’ is revised accordingly.

## Lessons learnt and future directions

Social media can be an invaluable tool to support the sharing of information. It plays a crucial role in disseminating the aim of iHealthFacts and the claims we have addressed. At a time when individuals were vulnerable and communities were physically distanced, connecting to trustworthy evidence through social media proved vital. However, unreliable health claims spread through social media can cause harm.

iHealthFacts was born in response to the surge of misinformation and disinformation around COVID-19. It is a resource where the public can quickly and easily check the reliability of a health claim circulated by social media. We evolved and grew throughout this venture by, listening to, and acting on feedback from members of the public and other stakeholders and we continue to welcome further suggestions. In our information-focused society, members of the public will be faced every day with new health claims. Moving forward, iHealthFacts will continue to fact check online claims that are submitted by the public, with both COVID-19 and non-COVID-19 related health claims welcomed. iHealthFacts will continue to provide reliable, current evidence to the public in an accessible format.

## Key messages

- The aim of iHealthFacts is to help people think critically about health claims and make well-informed choices.
- The role of social media platforms in battling an infodemic and promoting the dissemination of robust evidence is critical.

## Data availability

No data are associated with this article.
