# Supplementary material for: Battling the COVID-19 infodemic in an Irish context: the role of iHealthFacts
Source: HRB Open Res. 2020 Nov 9;3:81. [Version 1] doi: 10.12688/hrbopenres.13174.1 (PMC8132478; doi:10.12688/hrbopenres.13174.1)
Supplement: Peer review history

# Peer review: Reviewer response for version 1

**DOI:** 10.21956/hrbopenres.14300.r29260

**Published:** 2021-05-18

**Authors:** Joel M Topf

**Affiliations:** 1 Department of Medicine, Oakland University William Beaumont School of Medicine, Rochester, Michigan, USA

In this Open Letter, Zaki, *et al*., provide a description of a health information site called iHealthFacts. iHealthFacts is an Irish initiative to combat misinformation on social media by providing researched and expert opinions on health advice being spread on social media.

 The authors correctly grasp the importance of fighting misinformation with reasoned, well written descriptions of the current state of the science regarding COVID-19. The iHealthFacts team is a group of professionals from diverse backgrounds. Their methodology is well described and intelligently thought out for the task at hand. 

 My primary concern with the letter is the other side of the social media coin. Having well thought out answers to health questions is important but actually getting mindshare and traction on social media is just as important. Does iHealthFacts have a following on social media? When iHealthFacts tweets or posts to Facebook, do they get likes, retweets, and shares? After reading the article I am left with the fundamental question of does their effort make a difference. I am disappointed by the authors failure to describe any metrics of engagement or even a time line of when iHealthFacts went online. The Novel Coronavirus infodemic was most intense in March and April of 2020 ( Google Trends). After that the intense public interest in the topic waned. It would not surprise me if the idea for iHealthFacts germinated during that peak interest but did not get deployed until after the infodemic subsided. 

 In summary, this is an interesting project and the letter is a well written description of the goals of the project, it will be interesting to see follow-up publications with data to see if the authors accomplished their stated goals.

Does the article adequately reference differing views and opinions?

Yes

Are all factual statements correct, and are statements and arguments made adequately supported by citations?

Yes

Is the Open Letter written in accessible language?

Yes

Where applicable, are recommendations and next steps explained clearly for others to follow?

Yes

Is the rationale for the Open Letter provided in sufficient detail?

Yes

Reviewer Expertise:

Social Media, Medical Education, Nephrology

I confirm that I have read this submission and believe that I have an appropriate level of expertise to confirm that it is of an acceptable scientific standard.

# Peer review: Reviewer response for version 1

**DOI:** 10.21956/hrbopenres.14300.r28418

**Published:** 2020-12-16

**Authors:** Fareeha Farooq

**Affiliations:** 1 Department of Biochemistry, Sir Syed Medical College for Girls, Karachi, Pakistan

This article titled "Battling the COVID-19 infodemic in an Irish context: the role of iHealthFacts " has highlighted the role of social media in spreading awareness about covid and other health conditions. During COVID we have especially seen both authentic information spread and other claims which have no scientific basis. This has created trouble for people in terms of not only physical health but also emotional and raised red flags. iHealthFacts will play an important role for public in making informed choices and this collaborative team will use social media platform more effectively in combating "infodemic".

Does the article adequately reference differing views and opinions?

Yes

Are all factual statements correct, and are statements and arguments made adequately supported by citations?

Yes

Is the Open Letter written in accessible language?

Yes

Where applicable, are recommendations and next steps explained clearly for others to follow?

Yes

Is the rationale for the Open Letter provided in sufficient detail?

Yes

Reviewer Expertise:

Medical education, plagiarism, scientific misconduct.

I confirm that I have read this submission and believe that I have an appropriate level of expertise to confirm that it is of an acceptable scientific standard.
